# Just How Prevalent are Peptide Therapeutic Products? A Critical Review

**DOI:** 10.1016/j.ijpharm.2020.119491

**Published:** 2020-07-03

**Authors:** Yajie Zhang, Hairui Zhang, Debadyuti Ghosh, Robert O. Williams

**Affiliations:** Division of Molecular Pharmaceutics and Drug Delivery, College of Pharmacy, The University of Texas at Austin, Austin, TX, USA

**Keywords:** peptide, approved peptide products, formulation, delivery, peptide injection, oral peptide, excipients

## Abstract

How prevalent are peptide therapeutic products? How innovative are the formulations used to deliver peptides? This review provides a critical analysis of therapeutic peptide products and the formulations approved by the United States Food and Drug administration (FDA), the European Medicines Agency (EMA), and the Japanese Pharmaceuticals and Medical Devices Agency (PMDA). This review also provides an in-depth analysis of dosage forms and administration routes for delivering peptide therapeutics, including injectables, oral dosage forms, and other routes of administration. We discuss the function of excipients in parenteral formulations in detail, since most peptide therapeutics are parenterally administered. We provide case studies of alternate delivery routes and dosage forms. Based on our analysis, therapeutic peptides administered as injectables remain the most commonly used dosage forms, particularly in the form of subcutaneous, intravenous, or intramuscular injections. In addition, therapeutic peptides are formulated to achieve prolonged release, often through the use of polymer carriers. The limited number of oral therapeutic peptide products and their poor absorption and subsequent low bioavailability indicate a need for new technologies to broaden the formulation design space. Therapeutic peptide products may also be delivered through other administration routes, including intranasal, implant, and sublingual routes. Therefore, an in-depth understanding of how therapeutic peptides are now formulated and administered is essential to improve peptide delivery, improve patient compliance, and reduce the healthcare burden for these crucial therapeutic agents.

## Graphical Abstract

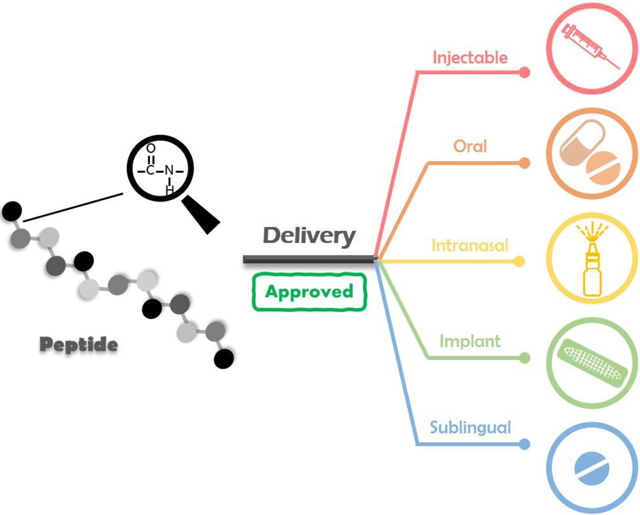

## 1. Introduction

*Peptides* are a class of molecules that fall between small organic compounds and large biological molecules (e.g., proteins) due to their distinct size, structure, and bioactivity. Peptides are considered as ‘building blocks’ of proteins, with smaller size and usually absence of higher order structures comparing to proteins [2]. Peptides are ubiquitous in cells and tissues throughout human body, where they are actively involved in vital physiological processes. Therefore, they are expected to be utilized as targets for diagnostic as well as therapeutic endpoints [3–7]. The global development, approval, marketing, and clinical use of peptide-based therapeutics has continuously increased in terms of market value and patient population size. Worldwide sales of therapeutic peptide drugs in 2017 was USD $20 billion, and sales are expected to surpass $50 billion by 2024, with a compounded annual growth rate (CAGR) of 9.10% during this timeframe [8, 9]. As of 2020, over 70 therapeutic peptide products have been approved as new chemical entities in the United States, Europe, and Japan, and more than 100 commercial products are available in the global market for various clinical indications. During the same time period, more than 160 therapeutic peptide products were reported in active clinical trials and more than 200 were reported in the preclinical development stage [8–12]. The indications of these therapeutic peptide products cover a wide range of diseases, including cancer, metabolic disorders, cardiovascular disease, respiratory disease, gastrointestinal disorders, skin diseases, central nervous system disorders, renal failure, hematological disorders, allergies, immune system deficiencies, liver disease, dental problems, and diseases of the eye [13, 14].

The debut of synthetic insulin in products for the treatment of diabetes introduced peptides to the realm of therapeutics in the 1920s. Before this, peptides were obtained only from natural sources: Insulin was derived from pancreatic extraction (circa 1920s) and adrenocorticotrophic hormone (ACTH) was derived from the pituitary glands of livestock (circa 1950s) [15, 16]. Synthesized oxytocin and vasopressin were approved for clinical use less than a decade after the introduction of the first ACTH–based therapeutic product. Their synthesized analogs also emerged, owing to the success of easier chemical synthesis of peptides [17–19]. The introduction of these peptide products highlighted the advent of a new therapeutic strategy. Unfortunately, further research in expanding the use of therapeutic peptides in the clinical setting decreased throughout the 1980s, partly because peptides lack the druggability of conventional low molecular weight drugs, and partly due to their extremely high production costs [20].

It is more challenging to formulate peptides as drug products compared to small-molecule drugs. Therapeutic peptides are more difficult to develop into dosage forms that are convenient for patients. The plasma half-life of peptides is usually very short (e.g., on the order of minutes for some unmodified peptides) due to the existence of several peptidases and the rapid renal and hepatic clearance rates in the human body [21, 22]. Peptides’ low permeability to cell membranes excludes the majority of lowlipophilicity peptides from targeting intracellular receptors and crossing the physiological barriers in absorptive areas such as the small intestinal tract. The poor oral bioavailability of peptides results from this limited absorption and from the significant first-pass metabolism inherent in oral administration, which results in enzyme- and pH-mediated hydrolysis in the gastrointestinal tract and liver [22]. Consequently, many peptides are formulated as injectable drug delivery formulations rather than oral dosage forms. This has a significant negative effect on patient compliance [23].

Nevertheless, therapeutic peptides are critical for future disease mitigation. Peptides retain the advantages of large molecules—i.e., high selectivity, high potency, and high specific binding affinity toward therapeutic targets [24]. As such, the typically less targeted compounds, such as small organic molecules, are not substitutes for peptides [25, 26]. For example, peptides typically exhibit superior capability for binding to and affecting receptors than their counterpart small organic molecules [14]. In addition, small-molecule drug discovery is more difficult due to large ligand-binding sites and the conformational changes required, for example in signal transduction of G-protein coupled receptors (GPCRs). As a result, peptides are becoming more favorable for GPCR–targeting agents compared to small organic molecules [14, 27, 28]. In addition to excelling in selectivity and potency, peptides and proteins also share the advantages of minimized systemic toxicity and low off-target side effects [20].

Due to the aforementioned features, and due to the fact that peptides and proteins differ essentially in the number of amino acids they possess, peptides are often mentioned along with proteins, but they are sometimes neglected. Yet, it is the difference in size and the resulting level of structure simplicity that differentiates peptides from larger proteins in certain aspects. For example, peptides have lower immunogenicity than proteins because they demonstrate less antigenicity [20, 29]. Moreover, the cost of peptide manufacturing is dramatically decreased as a result of advances in solid-phase peptide synthesis and purification strategies. Currently, over 85% of therapeutic peptides are chemically synthesized [22]. The pharmacokinetics (PK) and pharmacodynamics (PD) of peptides can be improved by incorporating modifications during their chemical synthesis to account for their disadvantages. The conjugation of entities, such as polyethylene glycol (PEG), lipids, proteins, and peptides have been demonstrated to effectively extend the half-life of peptides and improve their properties. As a result, this strategy is becoming increasingly prevalent in the development of therapeutic peptides [14, 30]. In addition, peptides’ intrinsic structural advantages and capacity for modification make them a preferable modality in terms of biological, chemical, and physical stability. Unlike therapeutic proteins, which are limited to injectable formulations and routes of delivery, therapeutic peptides are more flexible in terms of formulation and administration routes [31, 32].

**Figure 2.**
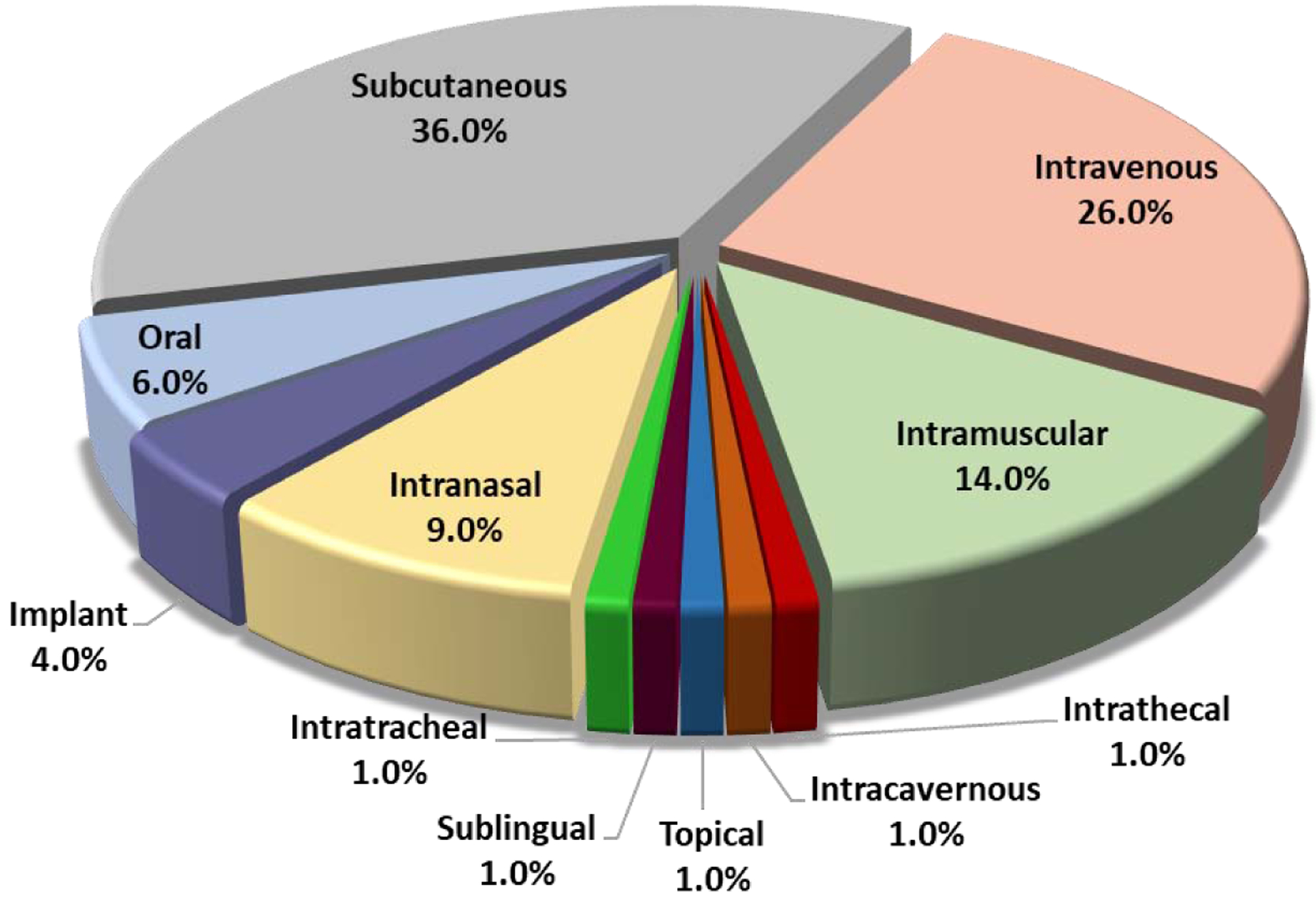
Distribution of administration routes for approved peptide therapeutics. Withdrawn products are also included in this statistical summary. The percentages refer to the number of products using the indicated administration route divided by the total number of product formulations. Some peptides are included multiple times because they occur in multiple products that use different routes of administration.

Eleven administration routes have been approved for therapeutic peptide products, including subcutaneous, intravenous, intramuscular, intrathecal, and intracavernous injections; implanted dosage forms; and oral, intranasal, intratracheal, topical, and sublingual delivery routes (see Figure 2). Injectable dosage forms are the predominant formulation type used for delivering peptides, comprising approximately 78% of approved administration routes. Amongst route of injections, subcutaneous injection is the most common, comprising almost 36% of total injections, followed by intravenous (~26%) and intramuscular injections (~14%). For the purpose of this analysis, *oral delivery* refers to administration via the gastrointestinal tract and does not include intraoral routes of delivery (e.g., sublingual, buccal). It is interesting to note that the intranasal route of delivery of therapeutic peptides was mostly utilized before the 1980s, but this has since fallen out of favor [11–14, 33, 34].

In this review, we will summarize and provide commentary on the excipients, formulations, and other technologies used in all peptide therapeutics that have been approved by the United States Food and Drug Administration (FDA), the European Medicines Agency (EMA), and the Japanese Pharmaceuticals and Medical Devices Agency (PMDA). All data and information have been updated as of January 2020. We collected data and information from a wide variety of sources, including the online databases of the three administrative agencies listed above, product labels, package leaflets, published scientific papers, patents, company websites, product websites, press releases, drug information repositories, conference abstracts, and conference presentations.

Our inclusion and exclusion criteria are adopted from Lau and Dunn [14]. We include peptides larger than two amino acids linked by one peptide bond, recombinantly expressed peptides less than 50 amino acids in length, and synthetic peptides of any length. Peptide conjugations are included as long as the peptidic portion meets the needs of the length limit [14]. We also include some peptide derivatives. When the same formulations are manufactured by different companies or provided in different doses, such formulations are counted only once. We exclude epitope-specific peptide vaccines, diagnostic-only peptides, peptide linkers, and peptides derived from non-recombinant bacterial fermentation.

## 2. Peptide Injectables

As demonstrated in Figure 2, parenteral routes are the most frequently used routes for the administration of peptide medications. The term *parenteral* refers exclusively to subcutaneous (SC), intravenous (IV), and intramuscular (IM) injections. These injections do not pass through the gastrointestinal tract, which protects peptides from the degradation induced by dramatic changes in pH and from numerous enzymatic digestion processes. As a result, peptides maintain their ideal bioavailability and efficacy. Additionally, injections avoid the limitations of peptides in regard to permeating biological membranes.

Peptide formulation scientists not only must meet the challenges and manage the risks of injectables in general, they must also solve the particular problems that occur in peptide injections. Generally, injection is not viewed as the best choice of delivery in most cases due to the inconvenience it poses for healthcare providers and poor patient compliance due to its invasive nature and the pain associated with injections. The need for special operations also increases the expense for both patients and health insurance providers. As mentioned in the introduction, the half-life of peptides is very short, so repeated doses are required within a short time interval, and this exacerbates the disadvantages. As a result, there is a strong demand for the development of modified-release formulations to resolve these problems. Moreover, injections bypass the body’s natural defenses and thus pose a greater safety risk. Consequently, injection formulations must be rigorously sterilized and must remain free of pyrogens and particulates during their shelf life (typically two years). The pH, injection volume, and tonicity must be appropriate for the injection route to prevent local irritation, discomfort, and hemolysis [23, 35, 36].

### 2.1 Physicochemical properties and stability of peptides

The physicochemical properties of therapeutic peptides are a barrier to formulating stable and effective dosage forms. The solubility of peptides depends on the pH of the solution, which results in minimum solubility at the peptides’ isoelectric point (PI). As such, pH should be adjusted to a value at which the peptide is completely soluble and in the tolerable range of injection (e.g., pH 3–10 for subcutaneous, intravenous, and intramuscular injectables) [32, 37, 38].

For injection routes other than intravenous, a higher concentration is usually required to deliver a certain amount of the peptide given the limited injection volume (typically considered 1.5 ml for SC) [36, 39]. This may introduce another peptide-specific concern: peptide self-association. Amphiphilic peptides tend to associate through interactions between the hydrophobic regions, which is enhanced as peptide concentration increases. As a result, the peptides in higher-concentration formulations may present in a highly associated state instead of as independent monomers. Theoretically, the peptide micelles formed from this behavior can enhance the peptide’s chemical stability due to diminished solvent accessibility. These micelles can also influence the peptide’s pharmacokinetics and bioavailability as well as its physical stability [40–43].

However, these molecular interactions can result in peptide aggregation, most commonly perceived as gelation and sometimes as precipitation [40, 44]. Other than self-association at high concentrations, peptides (unlike proteins) may exist in multiple conformations in solution rather than as a single stable secondary structure, and peptide conformations may change as the environment changes. Certain conformations are less stable and may lead to undesirable outcomes such as loss of function, increased adsorption, self-association, fibril formation, gelation (i.e., poor colloidal stability), aggregation, or precipitation [23, 45–47]. Therefore, the conditions of the formulation (e.g., pH, temperature, ionic environment) are critical to the physical stability of peptides.

In addition to physical stability risks, chemical stability should also be carefully considered when formulating peptides. Proteins usually have folded structures and thus prevent side chains from interacting with potential hazards. However, the typical peptide is fully solvent exposed, causing complete contact with reactive factors. Peptides containing oxidation-sensitive amino acids are particularly more susceptible. These amino acids include cysteine, methionine, histidine, tyrosine, and tryptophan residues [48, 49]. Light exposure can also accelerate oxidation, which is why the warning “protect from light” appears on most peptide product inserts. In addition to oxidation, peptides are susceptible to hydrolysis and deamidation due to the high degree of flexibility in the peptide chain and the lack of steric bulk in the amino acid adjacent to the carboxyl end of Asn [50]. Deamidation is more likely to occur when the pH is above 6, and it can occur in both solid and liquid peptide formulations. Typically, Asn–Gly, Asn–Ala, Asn–Ser, and Asn–Asp sequences have captured the attention of pharmaceutical researchers [40].

### 2.2 Formulations and Excipients in Approved Non-Modified-Release Peptidic Injection Products

**Figure 3.**
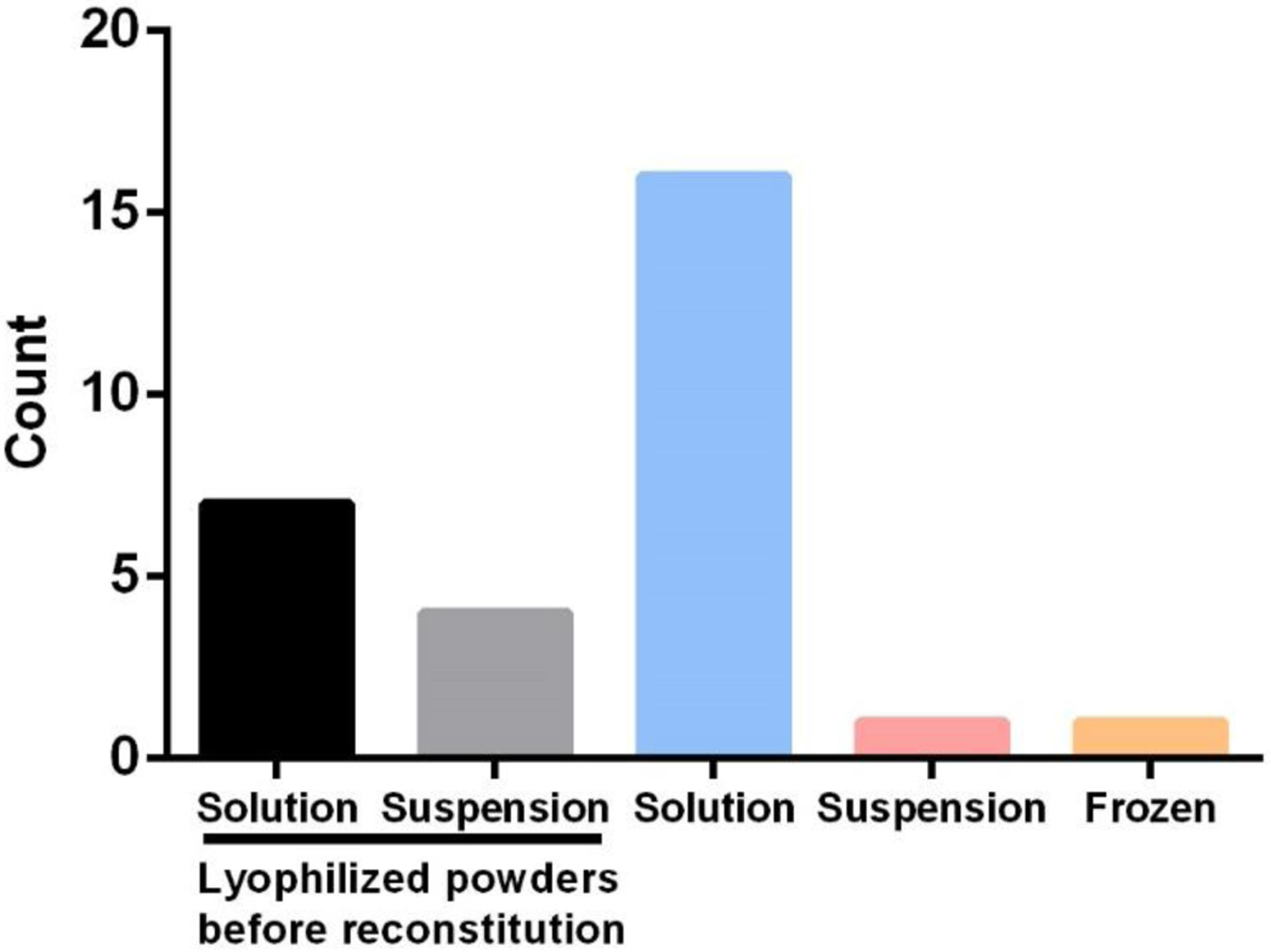
The number of forms of injectable peptide products that have been approved in the last 20 years and are not in discontinued status. The black and grey bars represent products in the solid state that are reconstituted either as a solution (black bar) or suspension (gray bar) before use.

The FDA, EMA, and PMDA have collectively approved more than 70 therapeutic peptide products since the 1950s, with more than 75% administered by injection (see Figure 2). Subcutaneous, intravenous, and intramuscular delivery are the most frequently developed routes. In contrast, only one intrathecal product and one intracavernous product have been developed. These products are manufactured and stored in the form of a lyophilized powder or in a frozen or liquid state (i.e., solution or suspension). Among the products approved in the last 20 years that have not been discontinued, over 55% are stored and injected in solution form (see Figure 3), while much of the remaining products are in a solid state as a lyophilized powder. Almost all these suspensions are modified-release formulations, and the majority of these are polymer-based formulations. Table 1 lists examples of non-modified-released injectable peptide formulations. The modified-released formulations will be discussed in the next section. It is important to note that long-acting peptides or peptide analogs (i.e., those that have a long half-life) are defined differently than modified-release products, whose prolonged effect depends on formulation technologies rather than modifications of the peptide molecule.

Generally, the pH of protein formulations are buffered in the range of 4.6–8.2 [32]. In addition, peptide formulations are composed of fewer categories of excipients than protein formulations. Protein formulations typically contain buffer agents, amino acids, stabilizers or bulking agents, surfactants, and tonicity agents. In contrast, typical peptide formulations exclude surfactants and amino acids, and they have a broader pH range. For instance, the pH of a reconstituted glucagon solution is as low as 2.5, while the pH of Fuzeon® rehydrated solution is as high as 9 [51–53].

In this review, we classify the excipients added to approved peptide formulations into seven categories, which are summarized in Table 2 below. The categories are *buffering agents*, *pH-adjusting agents*, *tonicifying agents*, *bulking agents or stabilizers*, *preservatives*, *antioxidants*, and *other* excipients.

#### 2.2.1 Buffering and pH-adjusting agents

The pH level of the solution plays a significant role in aqueous formulations of therapeutic peptides. The ability to maintain the formulation pH at a value that differs from the peptides’ pI enhances its solubility. Many physical and chemical degradation pathways are also pH dependent. In addition, a change in pH can cause the release of extractables and leachables from the container or closure of the products, leading to safety and stability risks. Furthermore, the pH of therapeutic peptide formulations must be adjusted to an acceptable range to prevent irritation, pain, or extravasation at the site of injection. As a result, in therapeutic peptide formulations, buffers and pH-adjusting agents are critical components that maintain a consistent pH over the product’s shelf life.

Buffer systems are typically composed of two chemical species that are related to a change in protonation state. When choosing buffer systems, the primary considerations include compatibility with other components of the formulation and the buffer capacity of certain salt concentrations. It is known that higher concentrations of buffer salts typically help improve buffer capacity. However, undesirable outcomes should be considered when increasing the buffer concentration. Some of these undesirable outcomes include the salting-out effect and crystallization-induced pH shifting upon freezing, as well as reactions at injection sites upon dosing. For example, it is reported that lower pH and higher buffer capacity are associated with increased myotoxicity of acetate buffers [68].

Table 3 lists the buffer systems deployed in approved peptide parenteral products, of which acetate is the most popular (> 50%), followed by citrate and phosphate. Acetate buffers has been so prevalent maybe because peptide purification processes commonly use acetate counter-ions as a substitute for trifluoroacetate (TFA) due to safety concerns. In addition to an acetate buffer, approved products such as Integrilin®, Natrecor®, and Kyprolis® contain citrate buffer species, while Invicorp, Victoza®, Ozempic®, and Gattex® are buffered by a phosphate system[56, 60, 63, 64, 69–71].

Salting-out salts, sodium phosphate, and sodium citrate were reported to be extremely effective in raising melting temperatures, which increases the shelf life of liquid protein formulations [72]. This may be an important consideration when choosing buffer systems for peptides that have higher-order structures. Moreover, citrate salts can remain in an amorphous state and minimize pH shifts before and after lyophilization, unlike sodium phosphate, which tends to crystallize and cause a pH shift of up to 4 units, possibly causing peptide degradation [73, 74].

Histidine is found in the phosphate-buffered lyophilized formulation Gattex®, which is most likely employed in order to minimize the pH shift provoked by freezing. Agents with a wide buffering range have been selected for use in liquid products such as Tymlos®, which uses succinic acid, and Signifor®, which uses tartaric acid[75, 76]. It has been reported that succinate and tartrate crystalize during lyophilization, which causes a pH shift. Tartaric acid can be amorphous in its buffering range (other than pH 3–4), while succinate crystallizes throughout its buffering pH range. Tartaric acid can also serve as an antioxidant in parenteral formulations [77]. In addition, some popular buffering agents in protein formulations (e.g., Tris buffer and glycine) are not yet found in peptide injection products. It has been noted, and widely cited, that Tris buffer degrades peptides by reacting with tyrosine residues and by liberating formaldehyde at a storage temperature of 70 °C [78].

Buffering species must be selected carefully in solid formulations [32]. Despite the above examples, the addition of buffer agents is not always beneficial or necessary for peptide formulations, especially for some peptides that can maintain a critical pH on their own. Products such as Cetrotide® (solid), GlucaGen® (solid), Copaxon® (solution), and Prialt® (solution) do not contain any buffer or pH-adjusting agents[57, 79–81]. Finally, hydrochloride and sodium hydroxide are typically applied in pH adjustments, even though acetic acid and sodium acetate are identified as pH-adjusting agents in some formulations, including Pitocin® and Symlin® [58, 82].

#### 2.2.2 Tonicifying agents

When developing injectable formulations, tonicity must be considered in order to create isotonic solutions for parenteral administration. Tonicifying agents are included in peptide injection formulations to ensure proper tonicity. This is because a non-isotonic solution can lead to tissue damage and increased pain at the injection site. Isotonicity in human plasma is equivalent to 290 mOsm/L. Therefore, this should be a target value for intravenous solutions or suspensions [86]. However, hypertonicity is actually required in some formulations. Wang, et al. reviewed the tolerability of hypertonic products, and they propose 600 mOsm/kg as the upper osmolality limit for intramuscular or subcutaneous injections and 500 or 1,000 mOsm/kg for intravenous and intravascular injections, depending on the injection volume [87].

Sodium chloride, mannitol, dextrose, and glycerin are specifically reported in product inserts as tonicifying excipients, as listed in Table 2. Sodium chloride is the most common tonicifying agent in peptide injection products. In several products, sodium chloride (NaCl) in various concentrations (e.g., 0.75% in Miacalcin®, 0.85% in Parsabiv®, 0.69% in Lutathera®) is added directly to liquid formulations[65, 85, 88]. Normal saline (0.9% NaCl) often serves as diluent and is absent in the freeze-dried powder of solid products. This is likely due to the fact that NaCl could greatly reduce the collapse temperature of the formulation, resulting in a low drying temperature and thus an increase in drying time and cost [89]. In addition, NaCl can not only crystallize on its own but also induce the crystallization of other excipients upon storage, which would impact the stability of the formulation [90]. Interestingly, the only frozen product, Bivalirudin in 0.9% Sodium Chloride® is frozen with 0.9% NaCl, which generates an osmolality of 300 mOsmol/kg for intravenous injection [55]. This could indicate that the impact of NaCl on freeze-dried product is during or after the drying stage. Freezing with NaCl does not necessarily harm the peptide.

Mannitol is demonstrated to be a suitable alternative to NaCl as a tonicifier. Mannitol often appears in subcutaneous solutions (e.g., Forteo®, Copaxone®, Signifor®), although only two pramlintide products, Symlin® and Byetta®, distinctly claim in their inserts that the purpose of adding mannitol is to adjust their tonicity [58, 76, 81, 91, 92]. Mannitol is frequently found in solid products as a bulking agent, some of which contain mannitol as their only bulking agent. The diluent for these products can be water for injection alone instead of isotonic solutions, which indicates that mannitol is not only a bulking agent in lyophilization but also a tonicifier for injectables. Examples of these products include Cetrotide®, Angiomax®, Firmagon®, and Egrifta® [59, 79, 93, 94]. Similarly, lactose, which is the only inactive ingredient in Glucagon®, may have the same use [53].

For injection, the selected diluents are normally water, normal saline, or 5% dextrose. It is worth mentioning that 0.9% NaCl is the diluent for Mepact, the only product that used a liposome delivery system[95]. Normal saline and 5% dextrose are used interchangeably for some lyophilized products, which gives both health providers and patients more practicality and flexibility. For example, Natrecor® lists 5% dextrose with 0.45% sodium chloride, USP, and 5% dextrose with 0.2% sodium chloride as alternatives other than 5% dextrose and 0.9% NaCl[70].

A pH-adjusted glycerin solution is the diluent of another glucagon peptide injectable formulation, Glucagon®[54]. In the reconstituted solution, glycerin (12 mg/ml) serves multiple purposes: It is a tonicifying agent and a cosolvent or solubilizing agent. It not only adjusts the osmolality of the solution but also helps the peptide dissolve in an environment with a pH between 3 and 9.5 [51]. In addition, solution injectable formulations, the liraglutide product Xultophy®, the bremelanotide product Vyleesi®, and both lixisenatide products (i.e., Soliqua® and Adlyxin®) used glycerin for tonicifying the formulation [62, 96–99]. It is worth mentioning here that glycerin is also a useful wetting agent for suspension formulations [77].

Propylene glycol is another common cosolvent or solubilizing agent. Propylene glycol is incorporated in the Ozempic® and Victoza® formulations, in which both semaglutide (the API of Ozempic®) and liraglutide (the API of Victoza®) are reported to be “good [*sic*] soluble in an aqueous solution” at their final pH [60, 71]. Thus, propylene glycol most likely serves as a tonicifying agent in these two formulations. Attention should be paid when using propylene glycol because it generates relatively high osmolality even in low working concentrations, despite being reported to inhibit gel formation of peptides and to has a wetting effect in suspension formulations [40, 100, 101]. The diluent for modified-release formulations will be discussed in the next section.

Achieving isotonicity can be challenging, especially for high-concentration formulations. Even though the acceptable range of osmolality is relatively broad, it is still worth noting that developers should alert healthcare providers and patients about the risk of injection-related pain under the “Adverse Reaction” or “Warnings and Precautions” section of the package inserts for formulations with high solute concentrations.

#### 2.2.3 Bulking agents or stabilizers

Sugars, sugar alcohols, and amino acids appear in both solid and solution peptide products as bulking agents or stabilizers (Table 4). They protect peptides and proteins from aggregating in liquid forms via interrelated mechanisms, including the excluded volume effect, increasing water surface tension, preferential hydration at high concentrations, or increased melting temperature (T_m_), which usually applies to larger peptides that have a complexed structure. In dry-state formulations, peptide structure can be perturbed by crystallization during freezing or by the removal of water from the vicinity during drying. This results in physical and chemical degradation. Sugars, sugar alcohols, and some amino acids can help peptides withstand these stresses and preserve their activity [72, 102, 103]. In addition to working as a stabilizer, some sugars (or sugar alcohol) and amino acids can act as bulking agents in a freeze-dried formulation matrix to ensure a desirable structure and appearance of the finished product. This is particularly important for formulations with low solid concentrations of substances (< 2%) [104].

Mannitol is the most extensively selected sugar alcohol excipient of approved peptide parenteral products in both liquid and dry states. As discussed in the previous subsection, mannitol also plays a role in balancing the tonicity of formulations. The strength of mannitol ranges from 4 mg/ml to 54.8 mg/ml in approved peptide products. In dry-state formulations, mannitol serves as a bulking agent because of its crystallinity, high eutectic temperature, and matrix properties [105]. These traits enable mannitol to maintain the structure of the lyophilized cake while simultaneously allowing drying under relatively aggressive conditions, which results in a shortened drying time and a lower cost [106, 107].

One should be careful when adding mannitol to a formulation, for a variety of reasons. First, although not been found in peptide formulations yet, it has been widely reported that the crystallization of mannitol during freeze-drying damages protein stability in both the presence and absence of amorphous excipients such as sucrose. Second, mannitol tends to form various crystalline modifications during storage, and this negatively impacts its storage stability [72, 108, 109]. Finally, impurities from mannitol supplies can induce oxidative degradation of a cyclic heptapeptide from a lyophilized formulations [110].

As discussed above, mannitol is found in almost all non-extended-released injectable formulations that contain sugars or sugar alcohols as their excipient, with the single exception of Glucagon®, which uses lactose[53]. Special attention should be paid when selecting lactose, glucose, or maltose, because their reductivity can lead to the Maillard reaction [111].

Histidine is a multifunctional additive in lyophilized bioproducts. Gattex® is the only peptide product that contains histidine[63]. Despite employing a phosphate buffer system in its formulation, histidine still likely acts as a buffer agent and a stabilizer rather than a bulking agent to mitigate the pH shift during lyophilization. This is because 7.76 mg/ml is not a typical working concentration for histidine as a bulking agent [32].

Sugars, polyols, and some amino acids are effective in stabilizing a variety of therapeutic proteins by protecting them from aggregation, denaturation, and other degradation pathways in both dried and solution states. As such, they are incorporated in almost every protein formulation [32]. Nevertheless, approximately half of peptide formulations are sugar free, particularly those formulations invented in earlier decades, such as Oxytocin®, Enalaprilat®, and Eptifibatide® [67, 112, 113]. This could be a result of the conformational simplicity of peptides compared to proteins, which require a higher-order structure (i.e., secondary, tertiary, and quaternary) to maintain activity. Sugars can preserve their structural integrity, and they ensure the protein remains in its native state through various mechanisms. The experience gained from protein formulation is valuable for stabilizing peptides, notably peptides with more complex structures. For example, other bulking agents and stabilizers (e.g., glycine, arginine, sucrose, sorbitol) can be considered as alternatives to mannitol.

#### 2.2.4 Preservatives

Antimicrobials are added to therapeutic peptide formulations, and they are required in multidose vials as preservatives to inhibit or reduce microbial growth. An antimicrobial is expected to satisfy several requirements. First, the antimicrobial must be effective against a broad spectrum of microbes, including bacteria, yeasts, fungi, and molds at low inclusion levels. Next, the antimicrobial must maintain its activity throughout the product’s manufacture, shelf life, and usage. In addition, the antimicrobial must have no influence on the quality or performance of both the active ingredient and other inactive additives. Last, the antimicrobial must have no adverse safety concerns [118]. Only a few antimicrobials have been approved for inclusion in pharmaceutical products, and even fewer are allowed to be used in parenteral products [119]. Only four preservatives have been identified in peptide injectable products: phenol, chlorobutanol, m-cresol (i.e., metacresol), and benzyl alcohol. Preservatives are usually added in small amounts (< 1%). Table 5 lists the working strengths of several preservatives documented in the literature or applied in approved peptide injectables.

Phenol and m-cresol are the most frequently used preservatives in liquid peptide formulations. Phenol is categorized as a phenolic compound and has a broad inhibiting effect on microorganisms, including mycobacteria, fungi, and viruses. The activity of phenol increases in acidic and concentrated solutions as well as at higher temperatures [32]. Aqueous solutions of phenol can be sterilized by dry heat or by autoclaving, but they should be protected from light exposure [119]. Another commonly used phenolic preservative, m-cresol, is found in later-stage developed peptide injectable formulations such as Byetta® and Soliqua®[92, 98]. M-cresol has been found to induce fewer adverse events. It is active against Gram-positive bacteria, but less active against Gram-negative bacteria, yeasts, and mold, and it is entirely inactive against spores.

Aside from phenol and m-cresol, chlorobutanol is an additive in most early-developed peptides such as vasopressin, oxytocin, and desmopressin. Chlorobutanol has a relatively low effective pH, as it is only stable at pH 3 in aqueous solutions. Increasing the pH results in a decrease in its antibacterial activity [120]. The pH of Vasostrict® (vasopressin), Oxytocin® (oxytocin), and Ddavp® (desmopressin) are 3.8, 4, and 4, respectively [66, 112, 121]. Chlorobutanol has bacteriostatic effects on both Gram-positive and Gram-negative organisms, and it exhibits some activity against yeasts and fungi [122]. Finally, benzyl alcohol is one of the most routinely added preservatives in parenteral protein formulations, and it is known for its less deleterious effect on protein stability. However, among all peptide injectables, benzyl alcohol is applied only in Enalaprilat®, in a concentration of 0.9% [67, 123].

Compatibility studies should be conducted when considering whether to add preservatives. This is a consequence of their reactivity with other ingredients, which can sabotage the activity of the active pharmaceutical ingredients and excipients. In addition, preservatives can also react with packaging and other materials during the manufacturing process [105, 119]. For example, Hejo et al. proposed that preservatives can adsorb oligomeric state peptides, and this interaction leads to both perturbed peptide self-interaction and impairment of antimicrobial efficiency [123].

#### 2.2.5 Antioxidants and Chelators

During manufacture and storage, three main sources give rise to oxidants in formulations: trace metal ions from containers or handling tools, hydrogen peroxide from sanitizing agents, and oxidant impurities from other excipients. Many amino acid residues in peptides and proteins are susceptible to oxidants, and they undergo chemical degradation when encountering them. Sulfur-containing amino acids (e.g., methionine, cysteine) are particularly sensitive to oxidation [124]. Metal ions (e.g., Fe^2+^ and Cu^2+^) can catalyze oxidative reactions in proteins or peptides through generating a redox oxygen species (ROS) either by binding directly to side-chain groups on the amino acids or by reacting indirectly with O_2_. The formed ROS can react with amino acid residues, which leads to peptide degradation [125–127].

Chelators and antioxidants are typically used to prevent the oxidation of active ingredients and excipients. Chelators stabilize proteins by forming multiple coordinate bonds with metal ions to reduce available ions for metal-catalyzed oxidative peptide degradation. Only two products, Invicorp (an intracavernous injectable) and Lutathera® (used in peptide receptor radionuclide therapy (PRRT)), incorporate the chelators disodium edetate (e.g., EDTA disodium salt) and diethylenetriaminepentaacetic acid (DTPA), respectively, in their formulations[56, 65]. EDTA is the most commonly used chelator in both chemical and biological drug formulations. It can chelate with metal ions, removing them from the solution in a process called *sequestering*. The working concentration of EDTA in parenteral formulations is reported to be between 0.01% and 0.2% (w/v) [77]. In addition to complexing with metal ions, EDTA can also be used synergistically as an antioxidative and as an antimicrobial with other antioxidants and preservatives [122]. The use of DTPA in Lutathera® is unique because the chelation of DTPA with metal ions not only stabilizes the formulation but also facilitates the clearance of free lutetium from blood via renal excretion, which subsequently reduces the risk of severe radiotoxic effects and other adverse effects [65, 128].

The addition of antioxidants can minimize oxidations caused by metal ions and other oxidation sources, such as trace impurities from other additives and hydrogen peroxide from sterilizing agents. Antioxidants can serve as electron donors to inhibit the oxidation of molecules in the formulation [129]. Methionine, ascorbic acid, and gentisic acid are included in approved non-modified-released peptide injection formulations. Prialt® and Soliqua® have incorporated methionine in their formulations, and Lutathera® contains ascorbic acid and gentisic acid[57, 65, 98]. Radiolytic degradation occurs frequently due to the high radioactive concentration of the therapeutic radiopharmaceuticals. The combination of ascorbic acid and gentisic acid act as free radical scavengers to provide better stability for the radiopharmaceutical products. Gentisic acid can be added to a formulation either before or after radiolabeling as long as the thermal decomposition caused by heating during labeling is within an acceptable range. Otherwise, post-labeling addition is more favorable [128, 130].

In addition to the molecules mentioned above, glutathione and several other amino acids (e.g., histidine, cysteine) also contain antioxidant properties. They are found in commercialized protein formulations, and they can serve as alternatives to methionine [32]. Acids such as tartaric acid also have antioxidative activity, but they simultaneously influence pH. Ascorbic acid can also serve as an antioxidant for non-radio-labeled molecules [77, 131].

#### 2.2.6 Other excipients

Two other excipients are identified in approved, non-modified-release peptide injectable formulations: sulfobutylether beta-cyclodextrin and zinc.

Sulfobutylether beta-cyclodextrin (SBECD), one of the polysubstituted derivatives of cyclodextrin, is employed in the peptidic product Kyprolis®, whose active peptide, carfilzomib, is a crystalline substance that is practically insoluble in water and only slightly soluble in acidic conditions [64]. Decreasing pH cannot achieve the desired concentration of carfilzomib, and researchers have found that this peptide is more prone to degrade in low-pH conditions. Therefore, the major responsibility of SBECD in a formulation is to solubilize the peptide to a necessary aqueous concentration without precipitation upon dilution. In addition to enhancing carfilzomib’s solubility, SBECD has been shown to provide isotonicity, and it acts as a bulking agent to support the integrity of the lyophilized cake [132].

Zinc is an additive in products that combine an insulin analog with a GLP-1 analog. The zinc ions in specific concentrations (55 μg/ml in Xultophy® and 30 μg/ml in Soliqua®, respectively) promote the association of insulin molecules in hexamers to increase the activity of proteins and improve both PK and physical stability [62, 98, 133, 134]. In addition, the formed complex results in delayed action of insulin [135, 136]. However, it has been demonstrated that the GLP-1 analog in Xultophy® (liraglutide) can form a zinc-binding liraglutide diheptamer in the presence of equimolar concentrations of zinc ions and diheptamers in stored insulin–liraglutide formulations[62]. It has been proposed that zinc-chelating compounds, such as histidine or imidazole, can be added to reduce the formation of liraglutide diheptamers [136].

### 2.3 Approved Modified-release Therapeutic Peptide Products

Long-acting formulations are frequently pursued for treating chronic diseases because they simplify the dose regimen and improve patient compliance by reducing administration frequency over the long-term health management period [137]. Even though proteins and peptides are highly potent molecules, they are constrained by their relatively short in vivo half-life and their poor bioavailability. These limitations result in the need for frequent and multiple doses, which leads to a poor quality of life for the patient, reduced compliance, and a loss of patient adherence to the treatment regimen. Aside from prolonging the half-life of a peptide by chemical modification, extended release can be achieved using formulation technologies such as nanoparticles, microparticles, peptide self-assembly, nanotubes, in situ gels, or a combination of different strategies [138]. These approaches enable peptides to be released in a specific pattern to improve the bioavailability and dosing regimen. The most prevalent delivery strategies for sustained-release peptides are implants and parenteral injections, primarily intramuscular and subcutaneous injections. These strategies enable the drug to avoid first-pass metabolism. In this section, we will focus on peptide injections; implants will be discussed in a later section.

#### 2.3.1 Polymer-based formulations

Approved PLGA–based peptidic products are usually provided in the form of lyophilized powder, and they are reconstituted to form a suspension before subcutaneous or intramuscular injection. The majority of approved modified-release peptidic products (e.g., Bydureon®, Triptodur®, Signifor Lar®) employ a polymer-based system, preferably a system based on a combination of polylactic acid and polyglycolic acid (a combination referred to as PLGA) [139–141].

PLGA is a general name for the family of biocompatible and biodegradable polymers composed of polylactic acid (PLA) and polyglycolic acid (PGA). PLGA usually refers to poly D,L-lactic-co-glycolic acid, where the D- and L- lactic acid forms are in a 1:1 ratio, unless specifically stated otherwise [142]. This polymer system has been extensively studied for delivering a variety of drug modalities, including small molecules, proteins, genetic therapeutics, and peptides, due to its favorable degradation properties, its sustained-release characteristics, and its versatility in achieving a desired dosage and release interval [142, 143]. PLGA degrades in aqueous media via hydrolysis of its ester linkage, which makes it biodegrade in an in vivo physiological environment. PLGA’s biodegradation rate is determined by several factors, including polymer composition, molecular weight, end groups, and the nature of the surrounding media. Given the same composition, higher molecular weights degrade faster than lower molecular weights. The monomer ratio of lactic acid to glycolic acid is critical in determining the degradation rate. The presence of the methyl group makes PLA more hydrophobic than PGA, thus systems with higher PLA ratio degrades more slowly because less water is available to trigger hydrolysis. However, the 50:50 ratio of PLA: PGA is an exception because it exhibits the fastest degradation rate [97, 100].

In the case of peptides, the PLGA drug delivery system dramatically extends the effective duration of the peptide formulations and consequently reduces the dosing frequency to a time scale of one month. Furthermore, a variety of timeframes is achieved by adjusting the PLA:PGA ratio or by adjusting the loading amount in the system. This allows more flexibility in treatment regimen design. As discussed above, the degradation time of the polymers greatly depends on their composition, such as the ratio of PLA to PGA. This is well reflected in the correlation between PLGA composition and dosing intervals in the approved products (see Table 6). Generally, a PLA:PGA ratio of 50:50 is chosen for a release term of one month. Higher PLA content gives a longer extension of up to six months, regardless the half-lives of the API peptides.

Lupron Depot® is supplied for one, three, four, and six months dosing schedules, although the half-life of its active ingredient peptide (leuprolide) is only 16 minutes [144, 145]. The application of the PLGA system prolongs the release of the peptide. The length of the period of continuous sustained release depends heavily on the adjustment of the molecular weight of the polymer and the composition ratio of PLG to PLA. The microspheres made with PLGA (75/25)-14,000 and PLA-15,000 achieved one month and three months of zero-order release, respectively [144, 146]. In addition, multiple dose options for the same dosing frequency are available by means of altering the amount of peptides loaded into the PLGA system. This allows products to be developed for different indications, and it facilitates the dose adjustment based on responses and tolerability during the treatment. Signifor® LAR® is a good example, since it is provided in 10 mg, 20 mg, 30 mg, 40 mg, and 60 mg doses for a 1-month supply, and the recommended doses for its two major indications, acromegaly and Cushing’s disease, are 40 mg and 10 mg, respectively [141].

Other excipients commonly found in PLGA–based peptidic products include mannitol, carboxymethylcellulose, polysorbate, buffering agents, and pH-adjusting agents. As in non-modified-release formulations, mannitol is a popular excipient that is widely employed in products. Mannitol is either added in lyophilized formulations as a bulking agent to prevent the aggregation of microspheres during the freeze-drying process, or it is added to diluent solutions to help the resuspension of the microspheres before administration [147, 148]. One exception is the group of exenatide products, which uses sucrose instead of mannitol, most likely as a protective agent [149]. Carboxymethylcellulose (CMC) sodium salt is added to the diluents of several products, including Lupron Depot®, Trelstar®, and Signifor® LAR® [141, 145, 150]. It provides viscosity to the system and stabilizes the suspension. Surfactants (e.g., polysorbate 80, polysorbate 20, poloxamer 188) are also routinely incorporated in diluents for wetting purposes. Moreover, the diluent of Bydureon® is buffered by a phosphate system, while other diluents adjust pH using acetic acid [139]. Some other excipients are selected for specific products. In another exenatide product, Bydureon Bcise® (a medium-chain triglyceride (MCT) suitable for use in an autoinjector) functions as a non-aqueous vehicle, which consequently generates an oily suspension [139, 151]. Gelatin is added in leuprolide formulations to increase encapsulation efficiency because of the high viscosity it generates in the primary emulsion [145, 147].

PLGA is a well-developed system for prolonged-release formulations. It is also the most widely used platform for modifying the release of peptidic drug molecules. As such, it is a good reference for formulation scientists. However, the PLGA system has some inherent disadvantages that must be managed. One problem is that microsphere-based formulations usually cause pain in intramuscular injections, and they require large injection volumes. This can limit the dose administrated at one time and can consequently shorten the interval of injections. Second, toxic organic solvents are involved in the manufacturing process, which could cause safety problems. The generation of acidic metabolites induces the risk of peptide degradation and increased local acidity, as well leading to irritation at the depot site. Third, as with all modified-release formulations, burst release and incomplete release should be major concerns. A high initial burst is a problem unique to the PLGA system when used for peptide delivery. In addition, the compatibility between the polymers and the peptide must be examined in pre-formulation [157, 158].

#### 2.3.2 Other Sustained-released Formulations

Somatuline Depot® (lanreotide) is the first marked sustained-release formulation produced via peptide self-assembly. This formulation is provided in a single-dose, prefilled syringe attached to an automatic needle guard system for deep subcutaneous injections [159]. Upon injection, lanreotide forms a depot at the injection site, and the precipitated drug is continuously discharged into surrounding tissues from the depot, likely through passive diffusion, followed by absorption into the blood. After a single administration, lanreotide is continuously released from the depot with a half-life of 23–30 days, and the achieved bioavailability in healthy subjects is up to 78.4% (for a 120 mg dose) [159].

**Figure 4.**
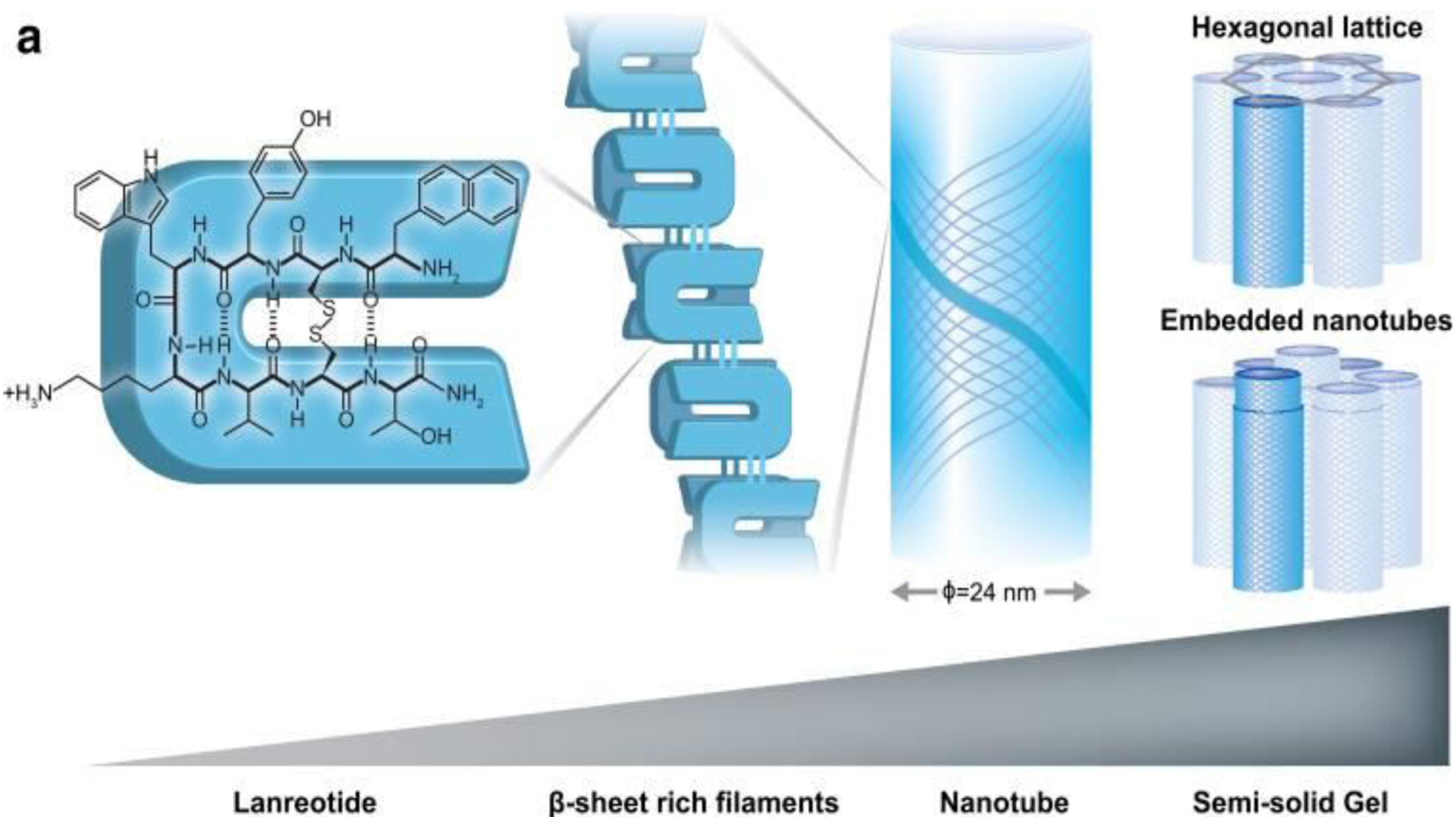
Lanreotide is a cyclic octapeptide that can form noncovalently bonded dimers. The dimers constitute β-sheet-rich filaments that are stabilized by hydrogen bonding. The hollow nanotubes are composed of 26 filaments, which have a highly uniform diameter of 24 nm. Depending on the lanreotide concentration, nanotubes in the semi-solid gel can be organized in a hexagonal lattice or they can form densely packed tube-within-a-tube structures (i.e., embedded nanotubes).

Wolin et al. have thoroughly discussed this product in their review paper, and the sustained release mechanism of the lanreotide depot has been sufficiently addressed [160]. Briefly, as illustrated in Figure 4, the cyclic octapeptide noncovalently dimerizes (mainly through hydrogen bonding), forming building blocks for β-sheet-rich filaments that constitute hollow nanotubes with a highly uniform diameter and wall thickness. A very dense packing of these constructed nanotubes creates a semi-solid gel structure. These nanotubes primarily form an organized hexagonal lattice with lanreotide concentrations of up to 10–15 % (w/w). However, at higher peptide concentrations, such as 24.6% in the product, a densely packed embedded tube structure begins to form. These nanotubes are also constructed by noncovalent forces such as hydrophobic interactions and π–π stacking of the aromatic side chains within the filaments. Consequently, in vitro experiments have revealed that the nanotube assembly is reversible, which enables the release of the drug [160–163].

It has also been noted that pH is critical for the formation of the depot. Acetic acid is the only excipient in the formulation, and its purpose is to maintain the pH at a level that ensures the injectability of the formulation and the solubility of the lanreotide [164].

Acthar® Gel is one of the first approved therapeutic peptide products (approved in 1952) that is still available. The active ingredient is adrenocorticotropic hormone (ACTH), which is eliminated rapidly with a half-life of around only 15 min [165]. It is administered via intramuscular or subcutaneous injection twice daily or once daily depending on the indication and the medical condition. The prolonged release is achieved by formulating ACTH with 16% gelatin. It is reported that the hydrolyzed gelatin counteracts the tissue inactivation phenomenon and enhances the stability and activity of the ACTH [166]. In addition, 0.5% phenol and no more than 0.1% cysteine are added as a preservative and an antioxidant, respectively. The pH is adjusted using sodium hydroxide and/or acetic acid [165].

## 3. Oral dosage forms of therapeutic peptides

### 3.1 Challenges

Oral delivery is the preferred administration route due to its convenience and because it improves patient compliance. However, the development and commercialization of oral-dosed peptide products has been hindered by the inherent drawbacks of oral dosage forms and by the properties of the peptides themselves. For example, the solubility and stability of peptides are generally sensitive to changes in pH, and a dramatic pH change occurs as the peptide moves through the gastrointestinal (GI) tract. Second, peptides are susceptible to digestion catalyzed by the rich enzymes along the tract, despite the fact that enzymatic products are sometimes tested for pharmacological activity because they might be active to a disease target [22]. Third, the presence of efflux pumps and a potential absorption limit are factors that inhibit peptides from crossing the epithelial barrier. In the Biopharmaceutical Classification System (BCS), peptides are usually classified as either BCS III (low permeability, high solubility) or BCS IV (low permeability, low solubility), indicating peptides’ generally poor permeability across epithelial membranes [21]. This poor permeability may exhibit a negative food effect in absorption. As a result, the evaluation of the food effect should be a necessity for oral peptide drugs. Moreover, some peptides are inclined to self-assemble to form fibrils, micelles, and aggregations, all of which decrease absorption. Even if it is going to be absorbed, the peptide must undergo the first-pass effect, after which systemic availability of the delivered peptide is dramatically decreased [23, 167–169].The poor bioavailability of peptides resulting from the reasons described above leads to a drastically higher dose in oral dosage forms than in injectables. This increases the already-high cost of peptide products.

The development of oral oxytocin and calcitonin demonstrates many of the difficulties of peptide drug development. Oxytocin is a cyclopeptide hormone that acts on the smooth muscle of the uterus to stimulate contractions. The product was initially approved in the 1960s, and it has been commercialized in the forms of intramuscular and intravenous injectables, as well as the currently discontinued intranasal dosage forms [33]. The oral bioavailability and efficacy of oxytocin and its analogs were widely evaluated in both human and animal models, yet the results demonstrate that the response of one 200 IU tablet of oxytocin is similar to that of an IV infusion of 0.02 IU/min [170]. In addition, the plasma level of oxytocin is drastically lower than that of IV administration, and metabolites were later found in circulation [171].

Another example is the exploration of the oral delivery of calcitonin. In 2011, Novartis Pharma AG announced that it would cease pursuing the development of oral calcitonin for postmenopausal osteoporosis osteoarthritis after 11 years’ years of effort because the key end points had not been met in two clinical trials. The main reason for this failure was that the polypeptide, which is a chain of 32 amino acids, was degraded by gastric acidity and digested by the proteolytic enzymes in the stomach and intestines, even with Emisphere’s Eligen® drug delivery platform [172–174].

The approved oral peptide products have low bioavailability, regardless of whether they are delivered in solid or liquid form (see Table 7). It has been statistically proven that solution and tablet dosage forms are bioequivalent in enalapril and lisinopril, although from a biopharmaceutical perspective, a solution is preferred over a tablet or capsule since the solution skips the steps of tablet disintegration and subsequent dissolution before absorption [175].

The package insert of Desmopressin Acetate® oral tablets claims that its bioavailability is only about 0.16% compared to intravenous delivery, which is even lower (0.08%) in the results of some other studies[176]. Yet, despite its low bioavailability, the pharmacodynamic effects of oral desmopressin are similar in magnitude to the effects observed after intravenous dosing at night and during the first six hours after daytime administration [177]. The low bioavailability combined with favorable pharmacodynamic effects might benefit from the fact that desmopressin is a cyclic peptide. Cyclic peptide structures improve the peptides’ stability in the GI tract because cyclization provides conformation constraints and reduces the flexibility of peptides, thus increasing proteolytic stability and permeability [22, 167].

The oral bioavailabilities of linaclotide, plecanatide, and elobixibat are negligible, but it does not impact their function because their bioactive actions occur locally in the GI tract. Linaclotide and plecanatide are both agonists of guanylate cyclase-C (GC-C), and they have local effects on the luminal surface of the intestinal epithelium. The activation of GC-C ultimately leads to accelerated GI transit and increased release of intestinal fluid [178]. Elobixibat is minimally absorbed in circulation when taken orally; however, it acts locally in the lumen of the GI tract by binding to and partially inhibiting ileal bile acid transporter (IBAT) in the ileal mucosa [179]. Therefore, the low systemic bioavailability supports these three peptides’ mechanisms of action as a local GC-C agonist or inhibitor of IBAT.

### 3.2 Strategies to develop oral dosage forms of peptides

Because peptides are limited by the problems mentioned above, only a handful of peptides are currently approved in oral dosage forms. These include desmopressin, enalapril, lisinopril, linaclotide, plecanatide, and the recently approved elobixibat. They are formulated mostly for tablets and oral solutions or suspensions. Linaclotide is the only peptide available in capsule form. Table 7 shows all the approved and available oral peptides. For peptides like desmopressin that have multiple formulations, only one representative formulation is listed. As shown in Table 7, the excipients used in oral peptide products are the ones commonly employed in oral formulations for small molecules. These compositions usually include diluents, binders, disintegrants, lubricants, glidants, stabilizers, and coating agents. The function and the commonly used chemicals for each category of components are extensively described in the literature, so they will not be discussed in this review [122, 180, 181]. Given that only a small number of oral peptide products are still in the discovery and preclinical stages, new strategies for orally delivering peptides are urgently needed.

The performance of oral delivery of peptides can be improved via structure modification, including conjugating the peptide to macromolecules or by using prodrugs, cyclization, terminal protection, or amino acid replacement [182–184]. In addition to these methods, formulation is another option to optimize the efficacy of oral peptides.

The co-administration of enzymatic inhibitors or absorption enhancers has been widely investigated. Enzymatic inhibitors can minimize the catalysis of administered peptides in the GI tract by specifically inactivating the peptidases, which eventually enhances their oral bioavailability. Extensively studied clinical-relevant enzyme inhibitors include soybean trypsin inhibitors, aprotinin, puromycin, N-acetylcysteine, and bacitracin [182, 184]. For example, the co-administration effect has been tested for insulin with aprotinin, acarbose, 4-(4-isopropylpiper-adinocarnonyl) phenyl 1, 2, 3, 4-tetrahydro-1-naphthoate methanesulphonate (FK-448), and many other protease inhibitors. These results suggest that the combination can improve insulin absorption in the intestinal lumen of tested animals [185]. However, it is well known that these inhibitors have risks, including systemic toxicity shock, interruption of the normal absorption of dietary peptides, as well as hypertrophy and hyperplasia of the pancreas [184].

Co-administrating absorption enhancers is another widely investigated strategy to improve oral bioavailability. Surfactants, bile salts, phospholipids, fatty acids, glycerides, toxins, lectins, cell penetrating peptides (CPPs), and polymers are all reported to enhance the permeability of peptides across intestinal epithelial cells through different mechanisms [22, 182]. Thanou et al. found that the intraduodenal administration of octreotide acetate together with chitosan hydrochloride 1.5% w/v to pigs produces a 3-fold increase in drug bioavailability compared to the control group [186]. Another study demonstrated that the addition of CPPs and hydroxypropyl-β-cyclodextrin in insulin formulations increases the transportation efficiency up to 10 times compared to insulin alone [187].

Moreover, mucoadhesive polymers can also enhance the intestinal absorption of peptides [188, 189]. For example, other than promoting adsorption, as previously mentioned, chitosan and modified chitosan are also common types of *thiomers* that can increase the mucoadhesive properties of the mucus gel layer up to 140-fold. As a result, the generation of a steep concentration gradient across the epithelial barrier facilitates the passive uptake of salmon calcitonin and insulin [189]. There is widely published evidence that demonstrates the effectiveness of adsorption enhancers and mucoadhesive enhancers. Yet, like inhibitor enhancers, the clinical and commercial success of permeation enhancers has not been achieved, even though a number of clinical trials are ongoing [22, 190]. Like enzymatic inhibitors, safety concerns are the major challenge for developing permeation enhancers.

Another formulation-related strategy is to apply an enteric coating in oral peptide formulations. This coating can protect peptides against the acidic and enzymatic stomach environment, escorting the peptides to exit the stomach with minimal damage. The enteric coating then breaks down in the intestinal fluid, followed by disintegration and dispersion of the dose [191]. The dissolution of the enteric coating can be triggered by different mechanisms such as ionic change, pH response, time, bacterial or enzymatic degradation, depending on the properties of the enteric coating agent. This technology has been adopted in multiple oral platforms to formulate peptides, including the Transient Permeation Enhancer (TPE®) system for octreotide delivery and the Protein Oral Delivery (POD™) system for insulin delivery [192–194]. Nevertheless, the reliability of this strategy is questionable due to the risk of unexpected burst release or loss of release control. The reliability of this strategy is also reduced by variations in the gastrointestinal environment, such as changes or differences in microflora, enzymes, or physiological factors in individuals. Also, the toxicity and metabolism of the coating material should be rigorously evaluated [195–198].

Carrier systems have also been extensively explored for the oral delivery of peptides. Various strategies can enhance oral peptide bioavailability, including emulsion systems, hydrogels, and various types of particulate delivery systems [199–203]. Generally, these carriers, with or without surface modification, improve the performance of the drug by protecting peptides from enzymatic degradation and/or increase the absorption of the peptides through the intestinal barriers [204–206]. One example of this is the effect of salmon calcitonin (sCT) on blood calcium levels, which was 8.2-fold higher when sCT was encapsulated into a liposome coated with a thiolated-chitosan, compared to its free sCT solution counterpart [207]. Many similar cases have proved the superior potential of carrier-based oral peptide delivery [192, 199, 208]. However, more in vivo studies and clinical trials are needed to realize the translation of research to clinical use.

Several advanced platforms have been applied in oral peptide delivery, such as SmPhill® in cyclosporine delivery, TPE® in the development of octreotide capsules (conditionally trade named Mycapssa®), and the application of Eligen® in the delivery of oral salmon calcitonin and the peptide hormones GLP-1 and PYY[194, 209, 210]. These platforms usually involve multiple formulation strategies. In TPE®, peptides are combined with permeability enhancers and then enclosed in an enteric coating capsule. This approach results in protection from digestive enzymes and triggers the transient expansion of tight junctions between intestinal epithelium cells [194]. Several peptides have reached the clinical trials phase due to these designs. Building on these insights encourages more platforms that were originally intended for small molecules to be used in oral peptide development.

The oral delivery of peptides remains appealing but challenging due to the unique structural drawbacks of peptides. There is still an urgent need for new technologies. Their development relies on close collaboration between medicinal chemists and formulation scientists. In terms of formulation, potential options to overcome the current obstacles include the discovery and use of novel excipients, advanced delivery platforms, and safer and more effective co-administration reagents. Furthermore, prolonged release is anticipated to compensate for the short half-life of peptides because patients will likely prefer injections once every month over taking pills two or three times daily.

## 5. Nasal delivery of peptide products

As previously discussed, both peptide injections and oral dosage forms have drawbacks; notably, low patient compliance and poor bioavailability. These drawbacks were a driving force behind research on alternative administration routes. Intranasal peptide products were approved mostly in the earlier period of peptide development. These products include Miacalcin® (API: salmon calcitonin, approved in 1995 by the FDA), Ddavp® (API: desmopressin, approved before 1982 by the FDA), and Synarel® (API: nafarelin, approved in 1990 by the FDA) [226].

Even though fewer approvals have been granted in recent years, the nasal route is still a viable alternative for the systemic delivery of drugs. The systemic drug absorption of nasal doses results from the nasal cavity’s relatively large surface area, highly vascularized subepithelial layer, and rapid drug clearance. Additionally, the passage of drugs from the nose directly into systemic circulation avoids the first-pass effect of the liver [227]. Furthermore, intranasal delivery is a good option for delivering hormone peptides that function in the brain, since these molecules can transverse the olfactory epithelium to the central nervous system through areas unprotected by the blood–brain barrier [228–231].

In addition to the common problems related to delivering peptides (e.g., enzymatic susceptibility, limited permeability), the delivery of peptides via the nasal route is also hindered by the short mucociliary clearance time (~7 min). Peptides, especially poorly permeable ones, are prone to be wiped away within this short time frame [232]. Peptidal application is also limited by molecular weight because it is believed that smaller peptides cross the biological barriers in the nasal cavity more easily than peptides that have higher molecular weights [233].

The approved peptide nasal products are usually provided in solution in a special intranasal nonpressurized dispenser that delivers a spray containing a metered dose of peptides. Peptide nasal spray formulations are generally simpler, and their compositions are similar to those used in injection solutions. For example, hydrochloric acid and hydroxide are used to adjust pH, while citrate, acetate, phosphate or a combination of these buffers are employed to ensure the pH of the formulation is maintained within an appropriate range to avoid irritation (typically pH 4.5–6.5) [233]. Sodium chloride and sorbitol are employed in different formulations to modify the osmolarity, which can substantially affect the nasal mucosa and subsequently influence drug absorption and bioavailability. However, isotonicity is not always ideal for intranasal solutions in terms of bioavailability. It has been demonstrated that certain deviations in isotonicity can significantly enhance bioavailability [234]. Chlorobutanol (0.5%), as used in peptide injections, is added in one of the desmopressin intranasal formulations (Ddavp®) as a preservative[235]. In addition to these common agents in peptide solutions, benzalkonium chloride, which is a quaternary ammonium, is frequently applied to act as a preservative to inhibit microbial growth in formulations. However, better alternatives are needed given the reported adverse effects of benzalkonium chloride, which include reduced mucociliary transport, rhinitis medicamentosa, neutrophil dysfunction, and nasal discomfort such as burning, dryness, and irritation [236, 237].

Noctiva® (API: desmopressin) is the only nasal peptide emulsion product. Cottonseed oil and water are the two phases, and they form an oil-in-water emulsion in the presence of a combination of polysorbate 20 and sorbitan monolaurate. A permeation enhancer, cyclopentadecanolide, is also included in this formulation in a 2 % concentration to improve the passage rate of the peptide through the nasal mucosa and ultimately increase its bioavailability [238–240].

It is reasonable to conclude that adapting injection formulation strategies to nasal formulations can reduce development time, cost, and risk. However, formulation scientists must consider the peculiarities of nasal delivery. For example, the optimum pH and osmolarity of nasal formulations can differ from those in injectables, and these factors can significantly affect their bioavailability [241, 242]. An acidic environment (pH 4.5–6.5) is widely considered to be the acceptable range for nasal formulations [243]. Deviations in isotonicity enhance the intranasal bioavailability of salmon calcitonin roughly 4- to 5-fold [234, 244].

High viscosity is generally not desired in injection formulations; however, it can play an important role in the absorption of nasal delivery drugs by affecting their retention time in the nasal mucosa. Therefore, proper viscosity enhancers can be added to peptide nasal formulations, such as Avicel and hydroxypropyl methylcellulose (HPMC) [245].

Enzyme inhibitors can also be co-administered with peptides to reduce degradation, especially since enzymes are distributed in the nasal cavity and membrane [246]. In addition, the bioavailability of the peptides can be promoted by adding permeation enhancers and bioadhesive reagents (e.g., gels) to delay mucociliary clearance, even though their toxicity remains a serious concern [247].

In addition, nasal formulations should not contain an unpleasant odor or excipients that can stimulate the nerves in the nasal cavity. Aside from nasal sprays composed of solutions and emulsions, other types of formulations could be studied for peptide delivery, including nasal drops, nasal powders, nasal gels, and nasal inserts [233].

Last, a successful nasal product requires coordination between the delivery device and the formulation. The design of the metering, spraying, and container closure systems is critical in generating doses that deposit in the appropriate location and are accurate and reproducible [244, 248, 249].

## 6. Other dosage forms

Progress has also been achieved with the approval of peptide products in many other dosage forms, including topical, sublingual, intratracheal, and implant delivery forms. The only approved topical peptide, eledoisin (approved in the 1970s), has been discontinued and will not be discussed in this review [226].

**Figure 5.**
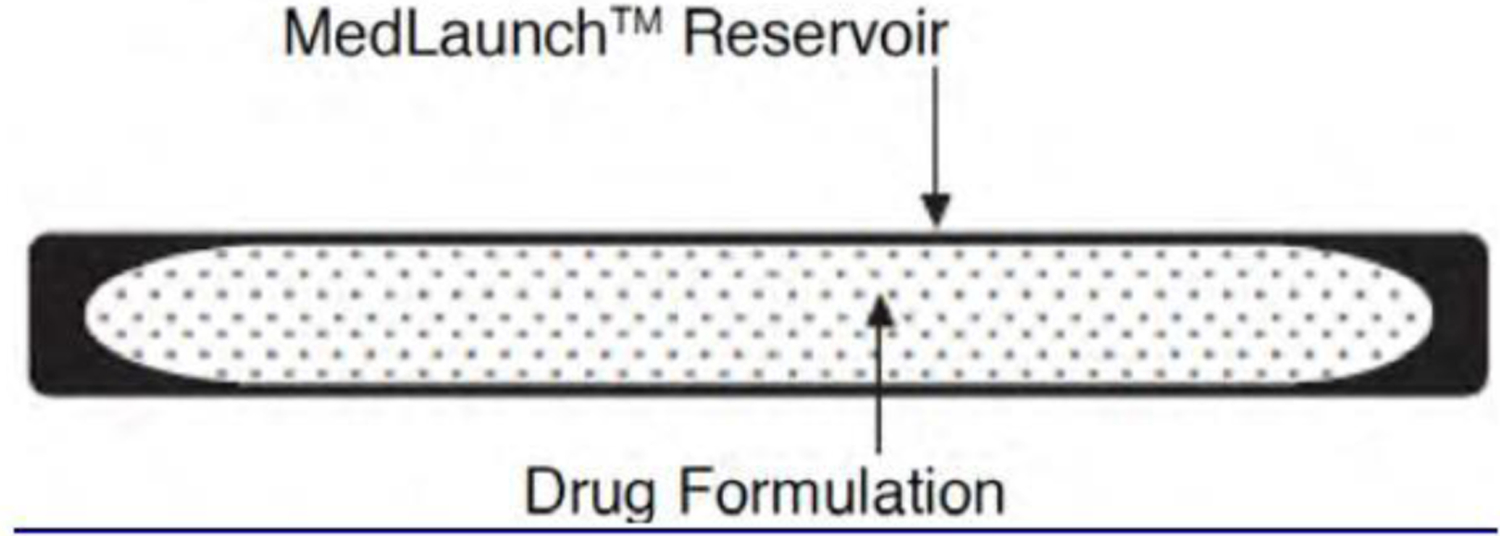
Supprelin® LA implant diagram (not to scale). Reprinted from the package insert of the Supprelin® LA implant [1].

Supprelin® LA and Vantas® are subcutaneous implants that contain histrelin. They are used to treat precocious puberty and prostate cancer, respectively [1, 250]. They employ the same techniques and formulation to consistently deliver histrelin at a rate of 65 μg/d over 12 months [1, 250]. The implant is constructed using Hydron® implant technology in which histrelin is formulated with stearic acid to form a solid drug core. This drug core is then placed in a hydrophilic cartridge reservoir called MedLaunch™ (see Figure 5), which is composed of 2-hydroxyethyl methacrylate (HEMA), 2-hydroxypropyl methacrylate (HPMA), trimethylolpropane trimethacrylate (TMPTMA), benzoin methyl ether (BME), Perkadox-16, and Triton X-100. A hydrogel with an equilibrium water content (EWC) of 29% can be generated by TMPTMA-mediated HEMA–HPMA copolymerization, which is optimized by the catalysts BME and Perkadox-16. Although not bound in theory, the diffusion of the drug occurs through a water channel in the hydrogel, and the drug release profile is manipulated by controlling the water content in the formulation. Another manipulative factor is the geometry of the drug delivery device, which in these products is a tube-shaped, 3.5 cm × 3 mm cylindrical reservoir. The implant is non-biodegradable, water-swellable, and water-insoluble [1, 250–252].

Scenesse® and Zoladex® are the two PLGA–based implants. Scenesse® has been recently approved by the FDA (approved in 2019). It contains afamelanotide as its API, and it is delivered by the PLGA system, allowing a dosing interval of two months. Afamelanotide (16 mg) is admixed with 15.3–19.5 mg PLGA copolymer in the implant core, forming a solid sterile rod approximately 1.7 cm long and 1.45 mm in diameter [253]. The ratio of PLA:PGA is most likely 85:15 (as reported in a related patent) [254]. Zoladex® contains goserelin acetate as its API, with a PLGA copolymer and acetic acid as inactive ingredients. The implant is prepared by dispersing goserelin acetate to a 50:50 PLGA system most likely in a 50:50 ratio (for four weeks of sustained release). This preparation forms a white to cream-colored cylinder 1 mm in diameter, which is then preloaded in a SafeSystem™ syringe [255, 256]. The PLGA system was discussed earlier in this review, and as such it will not be reiterated in this section. The main difference between Scenesse® and other peptide PLGA products is that the Scenesse® implant is injected as a solid rod instead of in a liquid or semi-liquid state.

The only sublingual peptide product, Nocdurna®, is developed as a tablet dosage form, delivering desmopressin for the treatment of nocturia due to nocturnal polyuria. This formulation contains only three inactive ingredients: gelatin NF (non-gelling), mannitol, and citric acid[257]. The gelatin is derived from fish sources, since its taste is less unpleasant than spray-dried mammalian gelatins. Fish gelatin is also preferred, since mammalian gelatin requires more heating time, and this increases the overall cost of the process. Gelatin functions as a carrier material by forming a rapidly disintegrable opening that ensures the matrix disintegrates in 10 s when it makes contact with the aqueous medium of the mouth. Mannitol is selected as the filler due to its fine particle size, which allows the tablet to dissolve readily in the mouth in seconds once it is wetted by saliva. Also, a fine particle size avoids a “gritty” or “sandy” texture, which gives patients an unpleasant organoleptic sensation. Citric acid is then added to help to achieve a pH of 4.8 [258, 259]. It is reported that the onset of antidiuretic action was observed within 30 min, and this effect persisted for 6 h after dosing with Nocdurna®. Despite the fast onset and the avoidance of both the hepatic first-pass metabolism and the degradation pathways in the GI tract, the bioavailability of Nocdurna® (0.25%) is only slightly higher than that of the desmopressin tablet (0.08–0.16%) [260].

The uniqueness in size and structure allows peptide formulation scientists to adopt inactive ingredients and process methods from both small molecules and biologics. Therefore, the development of more diverse dosage forms and formulations can be expected. For example, there are peptidic ophthalmic solutions, ophthalmic emulsions, topical gels, ophthalmic ointments, and inhalations in ongoing clinical trials [261]. Our team has successfully achieved caveolin-1 scaffolding domain peptide (CSP) inhalation formulations, including nebulization and dry powder inhalers [262, 263].

Yet, the uniqueness of peptides at the same time requires special treatment and considerations. For example, some salts that serve well in protein formulations may behave differently in peptide formulations because the thermodynamics of protein–salt interactions may not apply to peptides since peptides lack higher-order structures [264]. Another example is the use of ionization agents in synthesized peptides (usually acids such as trifluoroacetic acid (TFA) or acetic acid), which are routinely added during the late stages of peptide manufacture. The remaining ionic pair can impact the physiochemical properties of the peptide and consequently influence its formulation and biological properties, such as permeability across biological barriers. The residual acids can cause safety risks in addition to the influence in the physicochemical properties of formulations, such as solubility, crystallinity, and lipophilicity [265]. In inhalations, acids may cause an unpleasant odor and even irritation in the respiratory tract.

## 5. Conclusion

This paper serves as an overview of approved peptide products. It is intended to provide an update on the current state of the development of peptide therapeutics, and it offers practical reference materials for formulation scientists. The findings in this review reveal that the development of peptide drugs has achieved much success over the last century. Clinically available products continue to emerge, and practical knowledge of peptide formulation has increased alongside this growth.

However, novel excipients are anticipated to counterbalance the disadvantages of current routine excipients. New formulations and delivery platforms are needed to extend the duration of action to compensate for the short half-life of peptides. This would reduce healthcare costs and improve patient compliance. Most administration routes are injections (more specifically, IM and SQ injections), but the full diversity of peptide dosage forms should be explored, especially the less-invasive administration routes, to increase local effectiveness and patient compliance. Although it is challenging, formulation scientists will continue to pursue improvements in the oral delivery of peptides and the development of prolonged-release formulations. These approved peptidic products play a crucial role in the battle between medication and human diseases. We believe that, armed with a deeper understanding and with accumulated practical experience, more advanced excipients and delivery techniques will be invented. As a result, safer and more effective peptidic products will serve more patients in the near future.

## Figures and Tables

**Figure 1.**
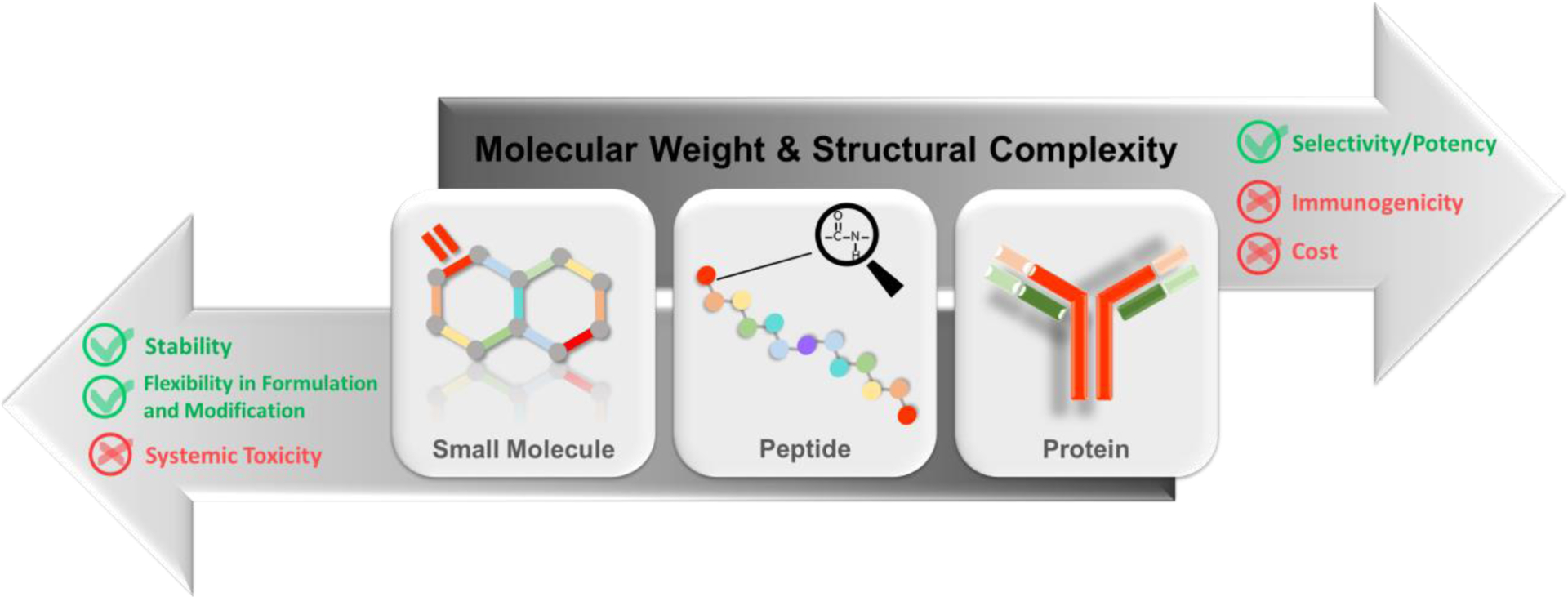
Peptides occupy the niche between small molecules and proteins.

**Table 1.** Examples of approved non-modified-release peptide injectable formulations.

Molecule	Brand name	Excipients	pH	Form*	Adm. route	Remark	Reference
Glucagon	Glucagon	Lactose and hydrochloric acid. Diluent: glycerin and water for injection.	N/A	Solid→ solution	SQ, IV, IM		[54]
Bivalirudin	Bivalirudin in 0.9% sodium chloride	Sodium chloride, sodium hydroxide and/or hydrochloric acid, and water for injection.	5.2–6	Frozen→ solution	IV		[55]
Aviptadil	Invicorp	Sodium chloride, disodium edetate, phosphoric acid, hydrochloric acid, sodium hydroxide, and water for injection.	N/A	Solution	Intracavernous	Combined with 2 mg phentolamine mesilate	[56]
Ziconotide	Prialt	L-methionine and sodium chloride. Diluent: 0.9% sodium chloride injection.	4–5	Solution	Intrathecal	Diluent must not contain preservatives.	[57]
Pramlintide	Symlin	Metacresol, D-mannitol, acetic acid, sodium acetate, and water for injection.	4	Solution	SQ		[58]
Degarelix	Firmagon	Mannitol. Diluent: water for injection.	N/A	Solid→ solution	SQ		[59]
	Victoza, Saxenda	Disodium phosphate dihydrate, propylene glycol, phenol, and water for injection.	N/A	Solution	SQ		[60, 61]
Liraglutide	Xultophy	Glycerol, phenol, zinc, and water for injection; hydrochloric acid and sodium hydroxide.	8.15	Solution	SQ	Combined with insulin degludec.	[62]
Teduglutide	Gattex	L-histidine, mannitol, monobasic sodium phosphate monohydrate, and dibasic sodium phosphate heptahydrate. Diluent: water for injection.	N/A	Solid→ suspension	SQ		[63]
Carfilzomib	Kyprolis	Sulfobutylether beta-cyclodextrin, anhydrous citric acid, and sodium hydroxide. Diluent: 5% dextrose injection.	3.5	Solid→ solution	IV		[64]
Lutetium Lu 177 dotatate	Lutathera	Acetic acid, sodium acetate, gentisic acid, sodium hydroxide, ascorbic acid, diethylenetriaminepentaacetic acid, sodium chloride, and water for injection.	4.5–6	Solution	IV	Peptide is radiolabled	[65]
Desmopressin	Ddavp	Sodium chloride, hydrochloric acid, and chlorobutanol.	4	Solution	IV, SQ	Chlorobutanol is added only in high-concentration formulations.	[66]
Enalapril	Enalaprilat	sodium chloride, sodium hydroxide, water for injection, and benzyl alcohol.	N/A	Solution	IV	Stored at 20–25 °C.	[67]
Abarelix	Fuzeon	mannitol, sodium carbonate (anhydrous), sodium hydroxide, and hydrochloric acid. Possible diluents: 5% dextrose injection, 0.9% sodium chloride, 5% dextrose injection in 0.9% sodium chloride, or 5% dextrose in Lactated Ringer’s Injection.	9	Solid→ solution	SQ		[52]

* *Form* refers to the packaged form (before reconstitution), *solid* means lyophilized powder, and *frozen* means frozen liquid. The symbol “→” represents reconstitution.

**Table 2.** Summary of excipients used in currently active non-modified-release peptide injection products.

Excipient class	Agent
Buffering and pH-	Disodium phosphate, monobasic sodium phosphate, and/or
adjusting agents	phosphoric acid Trisodium citrate and citrate acid Sodium carbonate Acetic acid and trihydrate sodium acetate Succinic acid Tartaric acid Hydrochloric acid Sodium hydroxide Histidine
Isotonicity agent	Mannitol Sodium chloride 5% dextrose Glycerin Propylene glycol
Bulking	Mannitol
agent/stabilizer	Lactose Histidine
Antioxidant	Methionine Ascorbic acid Gentisic acid
Chelator	Disodium edetate DTPA*
Preservative	Chlorobutanol Phenol Benzyl alcohol M-cresol
Other	Sulfobutylether beta-cyclodextrin Zinc

Note: Information is for current products in United States, Europe, and Japan that have not been discontinued. This information may not be comprehensive due to dynamic changes in the database and the authors’ accessibility to information.

* DTPA: Diethylenetriaminepentaacetic acid.

**Table 3.** Examples of buffer systems used in therapeutic peptide injection products.

Buffer system	Controlled pH range (25 °C)	Acid	Base	Example product	Product pH	Reference
Phosphate	5.8–7.8	Monosodium phosphate	Disodium phosphate	Ozempic	7.4	[71]
Acetate	3.8–5.8	Acetic acid	Sodium acetate	Ganirelix Acetate	5	[83]
Citrate	3.0–7.4	Citric acid	Sodium citrate	Integrilin	5.35	[69]
Carbonate	5.4–7.4,	Sodium	Sodium	Sandostatin	4.2 ± 0.3	[84]
	9.3–11.3	bicarbonate	carbonate	Fuzeon	9	[52]
Succinate	3.3–6.6	Succinic acid	Sodium succinate	Parsabiv	3.3	[85]
Tartrate	2.5–6.2	Tartaric acid	Sodium tartrate	Signifor	N/A	[76]

**Table 4.** Critical temperatures of bulking or stabilizing agents used in approved peptide non-extended-release injection products.

Agent	Function	Teu (°C)*	T_g_’ (°C)*	T_c_ (°C)*	Ref.
Mannitol	Bulking	−1.5	-	-	[114]
Lactose	Bulking/stabilizer	-	−28	−32	[115, 116]
Histidine	Stabilizer		−33	-	[117]

* T_eu_ refers to eutectic temperature (with water). T_g_’ refers to glass transition temperature. T_c_ refers to collapse temperature.

**Table 5.** Preservatives in approved peptide injection products.

Preservative	Reported Range in parenteral products	Range in approved peptide formulations	Example Product	Reference
Chlorobutanol	0.25–0.5 %	0.5–0.525%	Oxytocin®	[77, 112, 119]
Phenol	0.0715–0.5%	0.5–0.57%	Victoza®, Ozempic®	[60, 67, 77, 119]
Benzyl alcohol	0.75–5 %	0.9%	Enalaprilat®	[77, 119]
M-cresol	0.1–0.315%	0.22–0.3%	Byetta®, Soliqua®	[77, 92, 98, 119]

**Table 6.** Examples of PLGA–based sustained-released peptide injectable products.

Product	API	Half-Life of API	PLA: PGA	Dosing interval	Reference
Bydureon®	exenatide	2.4 h	50:50	1 week	[139, 152]
Lupron Depot®	leuprolide	3 h	75:25	1 month	[145, 147]
			100:0	3,4,6 months	
Trelstar®	triptorelin	6 min, 45 min, 3 h	50:50	1 month	[150, 153]
Sandostatin® Lar Depot	octreotide	1.7 h	55:45	1 month	[154, 155]
Signifor® Lar	pasireotide	12 h	50–60:40–50, 50:50	1 month	[141, 156]

**Table 7.** Oral therapeutic peptides and examples of their formulations.

API	Product	Dosage form	Oral bioavailability	Food effect	Target GI	Excipients	Ref.
desmopressin	Desmopressin Acetate®	Tablet	0.16% (to IV)	unknown	N	butylated hydroxyanisole, magnesium stearate, crospovidone, butylated hydroxytoluene, lactose monohydrate, povidone, and potato starch.	[176, 211]
enalapril (prodrug of enalaprilat)	Epaned®	Solution	38% (enalaprilat from oral enalapril	Y	N	citric acid, mixed berry flavor, purified water, sodium benzoate, sodium citrate, and sucralose; may contain hydrochloric acid or sodium hydroxide.	[175, 212– 215]
	Vasotec®	Tablet	maleate)	N		lactose, magnesium stearate, sodium bicarbonate, and starch. The 10 mg and 20 mg tablets also contain iron oxides.	
lisinopril	Qbrelis®	Solution	~25% (adults)	N	N	xylitol, sodium citrate, citric acid, sodium benzoate, and either hydrochloric acid or sodium hydroxide.	[216– 218]
	Lisinopril®	Tablet		N		2.5 mg: colloidal silicon dioxide, dibasic calcium phosphate, magnesium stearate, mannitol, pregelatinized starch and starch (corn); 5 mg, 10 mg, 20mg, and 30 mg: same with 2.5 mg but with the addition of red ferric oxide. 40 mg: same as with 2.5 mg but with the addition of yellow ferric oxide.	
linaclotide	Linzess®	Capsule	Negligible	Y	Y	72 μg: calcium chloride dihydrate, microcrystalline cellulose, L-histidine, polyvinyl alcohol, and talc. Capsule shell: gelatin and titanium dioxide. 145 μg and 290 μg: L-leucine, hypromellose, calcium chloride dihydrate, and microcrystalline cellulose. Capsule shell: gelatin and titanium dioxide.	[219, 220]
plecanatide	Trulance®	Tablet	Negligible	N	Y	magnesium stearate and microcrystalline cellulose.	[221, 222]
elobixibat	Goofice®	Tablet	Very low	Y	Y	*hydroxypropylmethylcellulose, talc, mannitol, carnauba wax, polyethylene glycol, crystalline cellulose, magnesium stearate, light anhydrous silicic acid, and a selection of titanium oxide, iron oxide, zinc oxide, tar pigment, and lake pigment.	[223–225]

* The reference of additives in Goofice® is patent US20170143738A1 instead of package insert, so it might not be the product’s final formulation.
